# Electromagnetic Differential Measuring Method: Application in Microstrip Sensors Developing

**DOI:** 10.3390/s17071650

**Published:** 2017-07-18

**Authors:** Francisco Javier Ferrández-Pastor, Juan Manuel García-Chamizo, Mario Nieto-Hidalgo

**Affiliations:** Department of Computer Technology, University of Alicante, P.O. Box 99, E-03080 Alicante, Spain; juanma@dtic.ua.es (J.M.G.-C.); mnieto@dtic.ua.es (M.N.-H.)

**Keywords:** microstrip sensor, dispersive media, differential measurement, remote sensing, multifrequency treatment

## Abstract

Electromagnetic radiation is energy that interacts with matter. The interaction process is of great importance to the sensing applications that characterize material media. Parameters like constant dielectric represent matter characteristics and they are identified using emission, interaction and reception of electromagnetic radiation in adapted environmental conditions. How the electromagnetic wave responds when it interacts with the material media depends on the range of frequency used and the medium parameters. Different disciplines use this interaction and provides non-intrusive applications with clear benefits, remote sensing, earth sciences (geology, atmosphere, hydrosphere), biological or medical disciplines use this interaction and provides non-intrusive applications with clear benefits. Electromagnetic waves are transmitted and analyzed in the receiver to determine the interaction produced. In this work a method based in differential measurement technique is proposed as a novel way of detecting and characterizing electromagnetic matter characteristics using sensors based on a microstrip patch. The experimental results, based on simulations, show that it is possible to obtain benefits from the behavior of the wave-medium interaction using differential measurement on reception of electromagnetic waves at different frequencies or environmental conditions. Differential method introduce advantages in measure processes and promote new sensors development. A new microstrip sensor that uses differential time measures is proposed to show the possibilities of this method.

## 1. Introduction

Electromagnetic (EM) waves interacts with matter in different ways. It depends on the radiation power, its frequency and the characteristic of the physical medium with which it interacts. In consequence, electromagnetic waves of different frequency interact differently with the medium through which they propagate. This interaction may be analyzed in the receiver measuring the parameters which characterize the signal (amplitude, propagation speed, phase, frequency, etc.). Matter is characterized by electromagnetic parameters (permittivity, conductivity, permeability) which can be used to detect it [1]. Different frequency and different matter parameters induce different interaction mechanisms: the medium can be analyzed measuring in the receiver after the interaction. This paper proposes an experimental approach to design sensors using these interactions and the theoretical model presented in [2].

This paper is organized as follows: Section 2 reviews related works (electromagnetic waves and its interaction with the medium). Section 3 proposes a new method which uses waves at different frequencies and measures using a differential method. In Section 4, advantages and disadvantages over the conventional scenarios are treated. In Section 5, experiments including software simulation of wave propagation dielectric medium are realized. In Section 6 a new microstrip sensor and differential measures are proposed. Finally, Section 7 provides conclusions and future works.

## 2. Related Work

Several disciplines exploit the interaction between electromagnetic waves and matter to detect and characterize the crossed medium. Biological systems or earth sciences that include the study of geology, the atmosphere, hydrosphere, biosphere, and the large-scale structure of the Earth’s interior use electromagnetic parameters like permittivity (ϵ), conductivity (ρ) and medium permeability (μ). Numerical values of these parameters characterize the analyzed medium. Frequency of electromagnetic wave is one of the main characteristics of the electromagnetic waves used in these applications.

Propagation speed, signal absorption and phase shift of electromagnetic waves in different transmissions depends on the radiation frequencies, and the electromagnetic parameters of the medium through which the waves propagate. These characteristics are used in different applications, measuring in receivers signals transmitted by wave emitters. In the study of geology, ground penetration radar [3] uses radar pulses to image the subsurface, the materials composition in the first layers of soil are detected analyzing its dielectric constant or relative permittivity (ϵ). Permittivity characterizes the analyzed medium and it depends on the frequency [4]. Different frequencies transmitted follow a procedure in which the emission, reception and analysis is realized. Effective permittivity of the material ($\epsilon_{eff}$) is calculated using the time of arrival ($t_{arr}$) of the electromagnetic wave in its reflection with the various layers which make up the subsoil. The arrival time of the signal depends on the frequency range (*f*) and the crossed medium, represented by its permittivity. The relations formed are arrival time ($t_{arr}$) function of frequency (*f*) and medium effective permittivity ($\epsilon_{eff}$).

In other scenarios like weather radars [5,6], the composition environment and variation in time are two aspects to analyze. Meteorological radars analyze the rainfall. In this case, the energy reflected in the rain drops are measure and analyzed. Here, the characteristics of medium analyzed is known (water) the objective is to calculate its volumetric density and its size in the form of a drop [7]. In this applications the relation between the energy received, working frequency and the rate of rainfall is analyzed [8]. Ionosphere analysis in [9] review the current state of knowledge of the European plasma environment with electromagnetic radiation and effects of the atmosphere on radio and radar signals is treated in [10].

Other important applications where effects of electromagnetic waves are critical are the biological systems. Understanding potential health and safety risks in medical applications and therapies [11] depend on the knowledge of the interaction with electromagnetic radiation. For a long time, the biological interaction medium-electromagnetic waves has produced numerous works [12,13], that use electromagnetic radiation with different frequencies, applications and biological scenarios. Electromagnetic waves are considered non-invasive methods that can characterize biological tissues [14]. Theoretical bases, experimental methods and practical applications analyze dielectric properties [15] using frequency spectra of electromagnetic radiation. Dielectric properties of the human skin is analyzed with new dielectric sensors configurations [16]. One of the latest trends in research for the construction of wireless sensors are microstrip patch sensors [17]. The basic idea of the microwave sensors is to measure the dielectric constant in a composite material (soil, water, biological, building structures) [18]. Microstrip configuration and coplanar circuits [19] are used in research works about human skin measurements during glucose excursions and wearable sensors.

The recent state of the art in physiological monitoring using wearable sensors, as review in [20,21], is the culmination of a decade of experimental developments from measurements in laboratory with instrumentation to design devices that permit continuous measurements. Accurate measures of the dielectric properties of the tissues does not depend only on these sensors, other requirements like sensor integration into the device, adapted user interface, data storage and analysis, pattern recognition and events with alerts are required [22].

## 3. Differential Measurement Model

**Figure 1:**
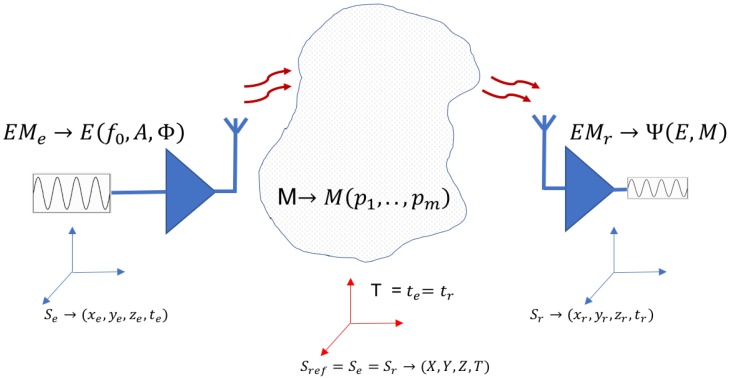
EM waves treatment in remote sensing applications. Devices need to synchronize the reference systems. Reference systems have the same reference time.

The interaction between EM radiation and medium analyzed uses positional and temporal reference systems $S_{ref}=S_{e}=S_{r}$ (Figure 1). Transmission and reception are calibrated and synchronized as a preliminary step to use in an application that characterizes the medium analyzed.

In geolocation systems, the position of triangulation devices and the synchronization of the clocks embedded in all devices is a necessary condition for addressing the task of determining the position of the receiver. In remote sensing applications, where the transmitter and receiver are not situated on the same device, temporal references (clocks embedded) must be synchronized. Once the necessary relation has been established between the referenced systems, a working EM with at a frequency band is used to induce the desired interaction and to obtain the information in the receiver. Calibration or synchronization of the system should be managed and maintained at all times.

The maintenance of these references in all devices has a cost which must be assumed into its functioning or operations. Electromagnetic wave emitted (*E*) is characterized by its Frequency ($f_{0}$), Amplitude (*A*) and Polarization (Φ). Signal received is a function (Ψ) that depends on signal emitted (*E*) and the medium, expressed by its parameters ($M=M(p_{1},\ldots ,p_{m})$). The function analyzed in the receiver is Ψ=Ψ(E,M) (Figure 1).

**Figure 2:**
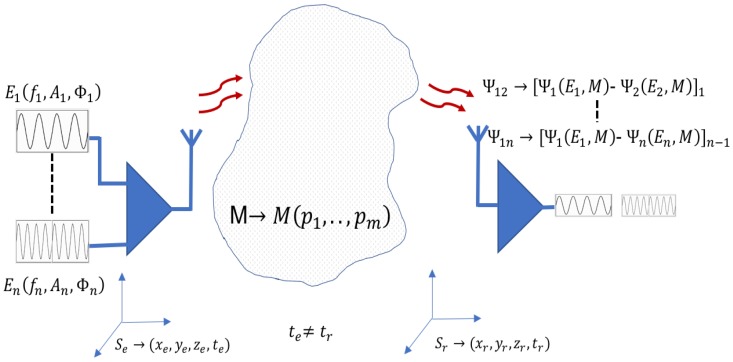
Differential method with different frequencies. The differential measures in the receiver incorporate information. Time synchronization is not necessary. The differential method proposed can also use a single frequency inducing different medium conditions.

Conventional treatment uses absolute measures on the receivers in synchronized reference systems. In contrast to this conventional signal treatment a differential measure model is proposed (Figure 2). In this new proposal, measured magnitude in the receiver (arrival time, signal amplitude, etc.) are relative. Differential measurements work with independent temporal references and enable the application of solutions with independent positional reference systems. Theoretical model is shown in Table 1.

In the differential measurement model, if the wave-medium interaction depends on the frequency and the medium, different frequencies will produce different measures in the receiver for the same medium and symmetrically, different medium parameters produce different measures for the same frequency. Using these differences, measured in the receiver, the parameters of analyzed medium can be identified. Differential treatment of the signal without time synchronization introduces operating advantages over the conventional treatment: it achieves some independence from the positional and temporal reference systems. Treatment with different frequencies and differential measures take advantage from information obtained on the interaction with each frequency, useful in the systems where the medium is parametrized. In addition differential measures compensate undesirable interference such as common interference or noises. With the aim of showing the differential method advantages, an example for positioning systems is analyzed below.

If the distance *d* between the transmitter and the receiver is known, different speeds of propagation using different signal frequencies or different propagation conditions can identify the parameters of the medium analyzed. This treatment could be used in applications where an unknown medium should be determined.

This work proposes to measure differences in the reception of EM waves. Two treatments can be used:

- If the wave-medium interaction is known, the link distance (emitter-receiver) can be estimated using differential measures in the receiver. This treatment is useful for location systems.
- If the distance emitter-receiver or some environment conditions are known, medium parameters can be estimated using differential measures in the receiver. This treatment is useful for medium detection systems.

In this paper, the work analyzes the model proposed and simulates differential measures to develop sensors in applications that detect dielectric mediums.

### Differences with Similar Methods

The differential method proposed uses similar techniques and technologies that already exist like stripline or microstrip [23]. In these methods electromagnetic transmission use absolute measures to calculate characteristic impedances. Hence, stripline or microstrip configuration reduces wave propagation speed ($v_{p}$) according to relative permittivity ($\epsilon_{r}$) of the dielectric substrate:

(1) $$v_{p}=\frac{c_{0}}{\sqrt{\epsilon_{r}}}$$

Likewise, the characteristic impedance in stripline and microstrip ($Z_{0}$) is dependent on geometrical dimensions (width, thickness and height) and the dielectric constant of the material used. If dielectric and dimensions are known, transmission processes are known, and vice-versa, if measures of transmission are known dielectric constant of materials can be characterized. In these and other similar cases, transmission and materials are analyzed using absolute measures of electromagnetic variables in a receiver device. The method proposed in this work uses striplines, microstrip or similar technologies extending its capabilities through a new model of utilization. In this way Equation (1) becomes:

(2) $$\Delta_{vp}=v_{p1}-v_{p2}=\frac{c_{0}}{\sqrt{\epsilon_{r1}}}-\frac{c_{0}}{\sqrt{\epsilon_{r2}}}$$

Basically, wave propagation speed ($v_{p}$) measured in conventional methods is replaced by differential speed measures ($\Delta_{vp}$), using similar techniques and configurations. Wave propagation speed is one of the possible differential measures. Losses, wave phases and others electromagnetic measures can also be used in a differential mode. To obtain differential measures, reference materials are used in new configuration sensors proposed in this method (being another difference in relation to the current systems).

## 4. Advantages and Disadvantages Over the Conventional Scenarios

Conventional scenarios in electromagnetic detection and identification can be characterized by measurement in the time domain. Time domain transmission (TDT) and Time Domain Reflection (TDR) measurements use test signals like step function and impulse. In these scenarios measurements are absolute and frequencies are selected depending on the process medium. Differential measurement has advantages over absolute measurement:

- A differential electric measurement is floating, meaning that it has no reference to ground. The measurement is taken as the voltage difference between two ports. The main benefit of a differential measurement is noise rejection, because the noise is added to both wires and can then be filtered out by the common mode rejection of the data acquisition system.
- The differential method proposed could use adapted TDT and TDR transmissions, expanding their capabilities on multi-frequency signals or multi-medium support.
- Conventional treatment must use a synchronized reference systems in the transmitter and receiver. In contrast to this conventional signal treatment a differential measure model is proposed and measured magnitude in the receiver (arrival time, signal amplitude, etc.) are relative. Differential measurements work with independent temporal references and enable the application of solutions with independent positional reference systems.

The main disadvantage of this method is that differential measurements are not easy to realize. Differential measuring method should be carried out by specialized equipment. A vector network analyzer could be used to analyze differences. Other instruments adapted to each kind of differential measure (phase, fields, impedances, etc.) must be developed to take advantage of this method in future works.

## 5. Simulation of wave Propagation in Dielectric Medium

The differential model uses different frequencies or different interactions induced by the medium through which the wave propagates. A dispersive medium (frequency dependent) is analyzed to determine the theoretical applicability. Dispersive medium are environments in which parameters like dielectric constant, permittivity or conductivity become dependent on the frequency. In this case, interactions in signal propagation like propagation speed or signal reduction (absorption) are frequency dependent. EM pulse propagation in different dielectric materials can be simulated using the numerical method Finite-Difference Time-Domain (FDTD) [24]. FDTD are full-wave techniques used to solve problems in electromagnetic that employs finite differences as approximations. This numerical method proposes computational algorithms (in this simulation Yee algorithm) to resolve the Maxwell equations in time and space domain. The FDTD formulation is a direct solution of Maxwell equations:

(3) $$\vec{\nabla }\times \vec{E}=-\mu \frac{\partial \vec{H}}{\partial t}$$

(4) $$\vec{\nabla }\times \vec{H}=\sigma \vec{E}+\epsilon \frac{\partial \vec{E}}{\partial t}$$

**Figure 3:**
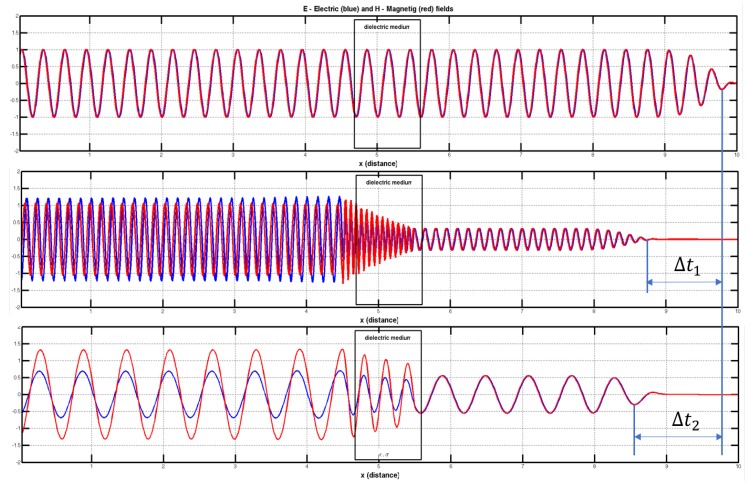
Multifrequency FDTD simulation in a dielectric medium. Transparent ($\epsilon_{eff}=1$) for the first frequency at the top. Different interaction (propagation speed and energy absorption) for other frequencies with different $\epsilon_{eff}$. Time differences $\Delta t_{1}$ and $\Delta t_{2}$ can be measured to analyze dielectric medium.

In Equation (3) the medium parameters are dielectric permittivity (ϵ), conductivity (ρ) and permeability (μ). This parameters represent the interaction of electric (*E*) and magnetic (*H*) fields. Finite Difference Algorithm is used to analyze electric and magnetic fields in space-time domain. A simulation using FDTD algorithm [25] is implemented in a numerical computing environment (MATLAB). Different simulations using this platform are performed to analyze differences between electromagnetic waves when arrive to receiver. Figure 3 shows the result in a dielectric and dispersive medium. This simulation shows how this differences are induced and confirm the first method hypothesis.

Figure 3 shows the effect that the model wants to use: different frequency produce differences in receiver. The simulations demonstrate a relation between time differences measured in receiver port and dielectric medium crossed. This first simulations with FDTD algorithms shows trends and effects of interaction with a general resolution. It serves to confirm the working hypotheses.

To show if differential measures can be used to take advantage of this interactions a simulation in a specialized software for EM interactions. This platform is Ansoft HFSS 13.0 software. This tool is used to simulate complex geometries using Finite Element Method (FEM) to compute electrical behavior of high speed and high frequency components. The HFSS most accurately characterizes the electrical performance of the components and effectively evaluates various parameters. Figure 5 shows the simulation results. One wave is transmitted within two parallel waveguides with different dielectric constant (ϵ) to produce different speed of propagation. One of the waveguides have ϵ=1 (air) and the other have an unknown dielectric material ($\epsilon_{r}$). Dielectric permittivity ($\epsilon_{r}$) of this material can be characterized using differential measures in the output ports.

It is assumed a medium with unknown dielectric permittivity ($\epsilon_{r}$) which is constant in a range of frequencies $\left[\omega_{m},\omega_{n}\right]$. An equation system to calculate ϵ is proposed. Dielectric Medium and wave speed, in dielectric medium, are related by Equation (5).

Equations system proposed to different ϵ are shown in Equations (5)–(7).

(5) $$\begin{array}{c} \epsilon \left(\omega \right)=\epsilon_{r}\epsilon_{0}\;\;\;\epsilon =\epsilon_{r}=>cte.\;\;\;in\;\;\omega_{m}<\omega <\omega_{n}\\ v_{p}=\frac{v_{0}}{\sqrt{\epsilon_{r}}} \end{array}$$

Waveguide long is L=60 mm, width (a=7.894 mm) and height (b=3.947 mm). The length of the dielectric material is $L_{\epsilon }=30$ mm. A broadband excitation pulse (1–10 GHz) is introduced on input waveguide port. Output ports receive signal at times $m_{1}$ and $m_{2}$. There are a $\Delta_{m}=\Delta_{t}=m_{2}-m_{1}$ obtained on output. This difference is used in Equation system (5).

(6) $$\begin{array}{c} m_{2}-m_{1}=\Delta_{t}=\frac{60\;\times \;10^{-3}}{v_{p2}}-\frac{60\;\times \;10^{-3}}{v_{p1}}\\ v_{p1}=v_{0}=3\;\times \;10^{8} \end{array}$$

**Figure 4:**
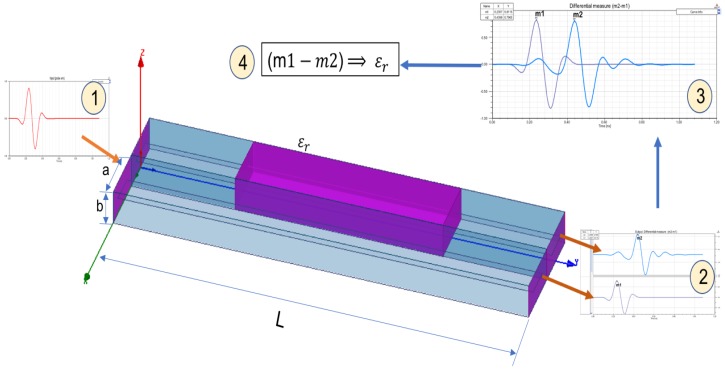
Rectangular wave guide (L=60 mm, a=7.894 mm and b=3.947 mm) used in propagation simulation built with two differentiated transmission lines. One input signal is transmitted (1) to the waveguide and derived into the two transmission lines. Two output ports signals are obtained (2) and time difference of arrival between two waves are obtained (3). The difference measured (4) characterize medium $\epsilon_{r}$.

Using the dimensions an data simulation (Figure 4), a relation between $\Delta_{t}=m_{2}-m_{1}$ measured and $\epsilon_{r}$ can be calculated. This relation is shown in:

(7) $$\sqrt{\epsilon_{r}}=0.1\;\times \;10^{11}\;\times \;\Delta_{t}+1$$

**Figure 5:**
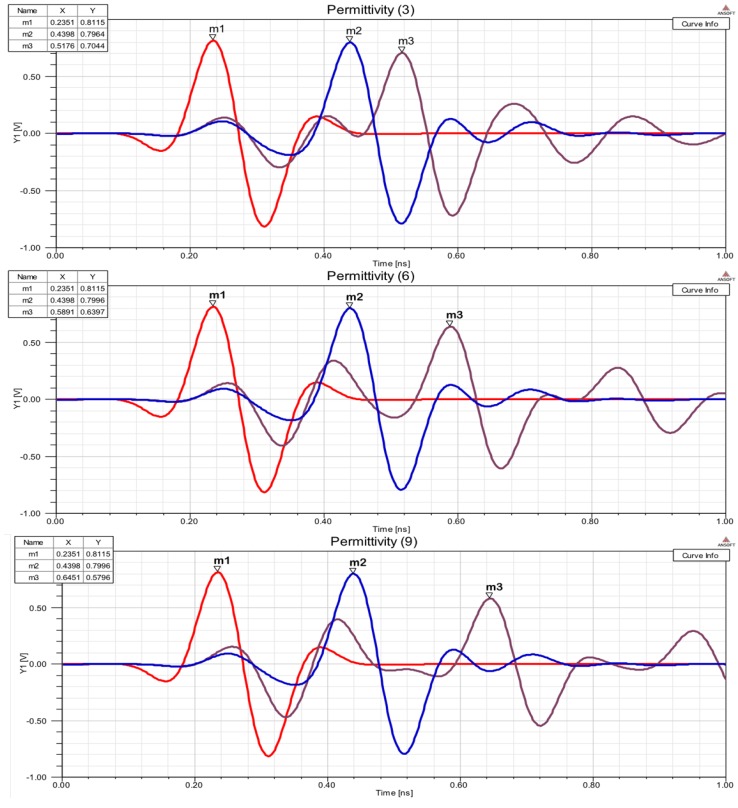
Three pictures with wave propagation simulation (in time domain) using waveguide shown in Figure 4; m1 is the input pulse, m2 is output line pulse with ϵ=1, m3 is output line pulse with unknown $\epsilon_{r}$. One input signal is transmitted (m1) to the waveguide and derived into the two transmission lines. Two output ports signals are obtained (m2 and m3) and time difference of arrival between two waves are obtained ($\Delta_{t}=m3-m2$). The measured difference (4) characterizes $\epsilon_{m}$.

Time analysis realized using electromagnetic wave transmission in rectangular waveguide, applying Equation (7) are shown in Table 2 and Figure 5.

**Figure 6:**
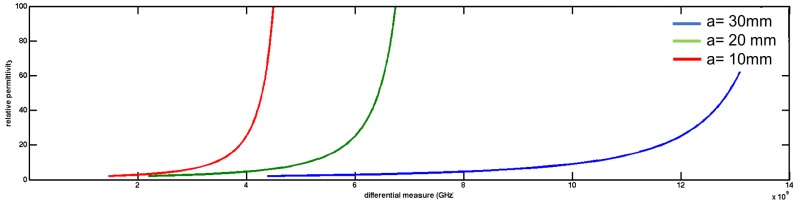
Permittivity obtained using differential measures in equation.

Air is the dielectric medium in line 1 (ϵ=1) and a dispersive medium unknown in line 2 $\epsilon =\epsilon_{r}$, with the waveguide walls conducting. In a rectangular waveguide is possible to propagate various modes of electromagnetic waves. Three modes of transmission TE (Transverse electric mode), TM (Transverse Magnetic mode) and TEM (Transverse Electric and Magnetic) are possible. In the rectangular waveguide supports TE and TM are supported and not TEM. A rectangular waveguide cannot propagate below a certain frequency which is called the cutoff frequency. The dominant mode in the rectangular waveguide considered is TE10 which has the lowest cutoff frequency. The cutoff frequency in a rectangular waveguide is given in Equation (8). The Figure 6 shows calculated permittivity using Equation (9).

(8) $$\Delta f_{c}=\frac{1}{2\sqrt{\epsilon \mu }}\sqrt{\frac{m^{2}}{a^{2}}+\frac{n^{2}}{b^{2}}}\;\;\;\;\;m,n=0,1,2,..$$

Different ϵ induces changes on $f_{c}$. Calculation and simulation results are shown in Table 3. Like in time domain, if $\epsilon_{1}$ is known in a transmission line, $\epsilon_{2}$ can be calculated using measures differences $\Delta_{f}=fc_{2}-fc_{1}$ in:

(9) $$\Delta f=\frac{1}{2\sqrt{\epsilon_{2}\mu }}\sqrt{\frac{m^{2}}{a^{2}}}-\frac{1}{2\sqrt{\epsilon_{1}\mu }}\sqrt{\frac{m^{2}}{a^{2}}}\;\;\;\;\;m=1,n=0,\epsilon_{1}=\epsilon_{r1}\epsilon_{0},\epsilon_{r1}=1,\mu =\mu_{0}$$

## 6. Microstrip Sensor and Differential Measures

Polymers, biopolymers, biological tissues, food products, porous materials, agricultural soil, plants, and others can all be considered as complex and dispersive systems. Measurement of the properties of dielectric has significant application areas like the aerospace, automotive, food industry, pharmaceutical, medicine, agriculture, defense industry and microwave device fabrication. Developments in the determination of material properties and their contribution to various industries for a wide range of applications are known [26].

Initially, sensors for detecting the properties of materials were waveguides and open-ended probes that were usually bulky and heavy [27]. Soon planar microwave components brought the possibility of usage of planar, small sensing devices that are used for characterization of a material. Rectangular microstrip patch antenna as a sensor for permittivity measurement is presented in [17]. A microwave sensor operating in the X-band (8.2 to 12.4 GHz) for detecting thickness or permittivity changes in one layer of multilayered dielectric structures is presented in [28]. A novel soil-moisture sensor based in a microstrip sensor placed in the soil is tested at 1.2 GHz in [29]. Tissue measurement using electromagnetic field and microstrip electrodes are analyzed in [17]. The latest references show that the dielectric materials can be specifically investigated and modeled by electromagnetic sensors with microstrip transmission. The current state of the art in continuous monitoring using electromagnetic wearable sensors, presented in [30], represents the design of broadband devices that permit continuous measurements in everyday life situations [15]. These sensors measure evolution and changes in dielectric constant and correlate these changes with the variation in blood glucose. By incorporating a number of sensors with differing geometric characteristics (microstrip dimensions), it is possible to show that a different penetration depth of the electromagnetic field into the tissue could be achieved.

**Figure 8:**
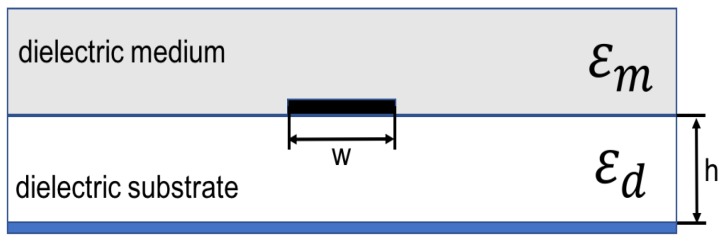
Basic microstip sensor.

In Figure 8a microstip sensor is shown. The influence of the material that must to be detected on signal propagation is reflected in effective permittivity of the microstrip, which depends on medium placed over miscrostrip (unknown material with $\epsilon_{m}$), and dielectric substrate (known $\epsilon_{d}$), from which microstrip sensor is made. Effective permittivity of the microstrip shown in Figure 8 is defined in Equation (10).

(10) $$\epsilon_{eff}=\frac{\epsilon_{m}+\epsilon_{d}}{2}+\left(\frac{\epsilon_{m}-\epsilon_{d}}{2}\right)\left(\frac{1}{\sqrt{1+12\frac{h}{w}}}\right)$$

**Figure 9:**
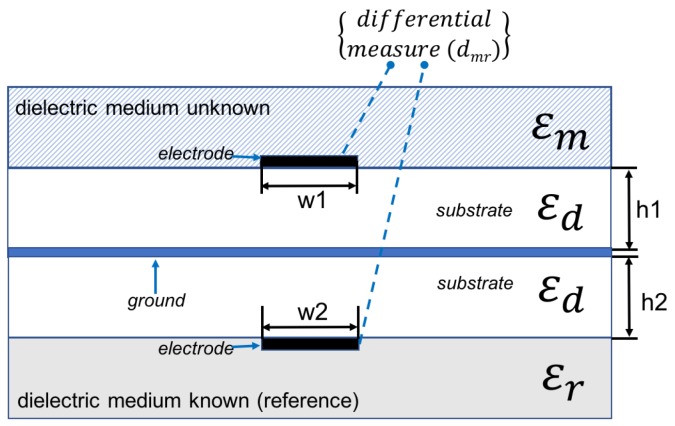
Microstip sensor proposed. Differential measures on electrodes are performed.

**Figure 10:**
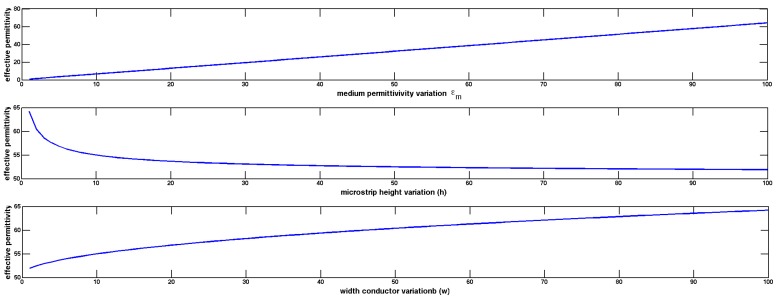
Sensitive analysis to determine how different values of dielectric medium ($\epsilon_{m}$) and microstrip dimensions (h and w) impact on effective permittivity ($\epsilon_{eff}$) shown in Equation (10).

In Figure 9, differential measure and microstip design is shown. In Figure 10, a sensitive analysis is realized. The aim is to obtain information of unknown medium with differential measures. The sensor used in this study has a combination of two electrodes of similar geometries separated and surrounded by a ground electrode. The electrodes are made of copper. The substrate material comprises of a standard dielectric used in circuit board, FR4 (standard circuit board material composed of fiber glass and epoxy resin). To complete the electromagnetic field simulations, different assumptions were made. These assumptions include the reduction of the 3D to a 2D simulated space. Since the lengths of the electrodes (60 mm) is larger than their width (2 mm), in any profile across the longer size of electrodes (except the electrode ends) the field distribution is simplified with the 2D distribution. It was shown in [31] that the simulation error caused by such reduction is smaller than 10%.

**Figure 11:**
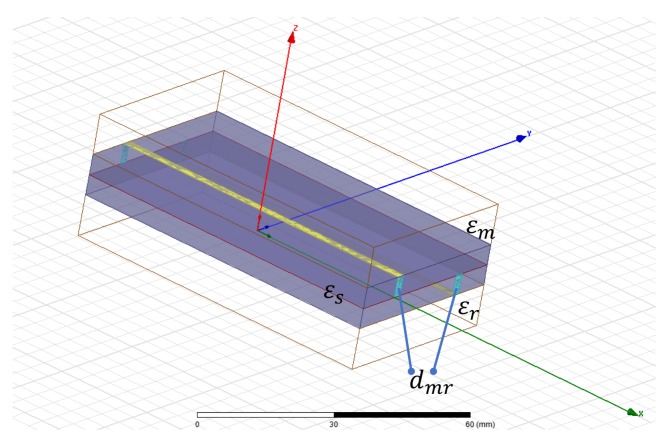
General model of microstip sensor proposed (L=25 mm, width=10 mm, height=2 mm and patch line=0.8 mm). Numeric differential measures ($d_{mr}$) can be obtained to characterize dielectric medium unknown ($\epsilon_{m}$), knowing reference permittivity ($\epsilon_{r}$) and microstrip substrate ($\epsilon_{s}$).

Theoretically, considering Figure 11 structure:

If $d_{mr}$ is =0 then $\epsilon_{r}=\epsilon_{m}$.

If $d_{mr}$ is ≠0 then $d_{mr}=f\left(\epsilon_{m}\right)$, $\forall \epsilon_{r}\neq \epsilon_{m}$, knowing $\epsilon_{r},\epsilon_{s}$ and microstrip dimensions.

A numeric simulation was realized using HFSS software (Figure 11) to test these assumptions.

Estimations of the wave phase incursion kl in the different sub-domains of the system sensor-medium where:

(11) $$k=\frac{2\pi f}{c}\sqrt{\epsilon }$$

*f* is frequency in Hz, $c=3\times 10^{8}$ m/s, ϵ is the dielectric permittivity of the sub-domain and *l* is its linear size. The electromagnetic wave propagation processes can be neglected if kl<<1 in selected frequency range, and thus the electromagnetic field can be assumed to have an electrostatic character. According to the electrostatic approach, the sensor coupled to skin and under-lying tissue can be considered as a lump capacitance element *C* in parallel with the resistance *R*. Thus, the description of the microstrip sensor can be reduced to an RCL resonant circuit. Parameters *C* and *R* are t unknown functions of these space distributions. The simplification of the sensor to a resonant circuit with lumped elements, allows to consider the sensor coupled to the medium unknown in terms of *C* and *R* parameters. Unknown medium are characterized by differential measure ($d_{mr}$). The influence of dielectric medium on signal propagation is reflected in effective permittivity of the microstrip, which depends on medium placed over miscrostrip, in this case a dielectric material known and other unknown. There are several differential measures in $d_{mr}$ that can show unknown material information:

- Differential phase shift
- Differential electrical field
- Other differential measures (impedances, signal loss, etc.)

Differential phase shift and electric field energy are analyzed in simulations on microstrip configuration shown in Figure 9.

### 6.1. Differential Measure Based in Phase Shift

A sinusoidal signal (Asinωt) is considered. This signal propagates along the transmission line in order to determine the impact of the physical parameters of the microstrip measuring phase shift. At the beginning of line, at the moment $t_{0}$, the phase of the signal is $\omega t_{0}$. At the end of the line, the moment is $t_{1}$, and its phase is $\phi =\omega t_{1}$. The phase (ϕ) at the end depends on electrical length ($L_{e}$), and the wavelength on dielectric medium ($\lambda_{eff}$):

(12) $$\phi =\frac{2\pi }{\lambda_{eff}}L_{e}$$

like propagation velocity in the medium ($v_{prop}$) and the wavelength ($\lambda_{eff}$) are :

(13) $$v_{prop}=\frac{c}{\sqrt{\epsilon_{eff}}}\;and\;\lambda_{eff}=\frac{\lambda_{0}}{\sqrt{\epsilon_{eff}}},\;where\;\lambda_{0}=\frac{2\pi }{w}c$$

then:

(14) $$\phi =\frac{2\pi \sqrt{\epsilon_{eff}}}{\lambda_{0}}L_{e}$$

Using microstrip line configuration (Figure 11) differential measure are:

(15) $$d_{mr}=\Delta \phi =\frac{2\pi \sqrt{\epsilon_{meff}}}{\lambda_{0}}L_{e}-\frac{2\pi \sqrt{\epsilon_{seff}}}{\lambda_{0}}L_{e}$$

(16) $$d_{mr}=\Delta \phi =\frac{2\pi }{\lambda_{0}}L_{e}\left(\sqrt{\epsilon_{meff}}-\sqrt{\epsilon_{seff}}\right)$$

Δϕ is measured at the end of the line, $\epsilon_{r}$ is a reference (known permittivity) and the $\epsilon_{m}$ is the unknown permittivity, calculated using HFSS simulation. The thickness of the dielectric substrate is 0.8 mm. The width of the microstrip line is w=2 mm, while the length is 25 mm. Substrate material is FR4_epoxy (with $\epsilon_{s}=4.4$ and a surface of 25×10 mm).

Numerical data in Table 4 shows the differential measures obtained at the end of microstrip line, considering frequencies between 1 and 8 GHz (rows) and a reference permittivity of $\epsilon_{reff}=4$.

**Figure 12:**
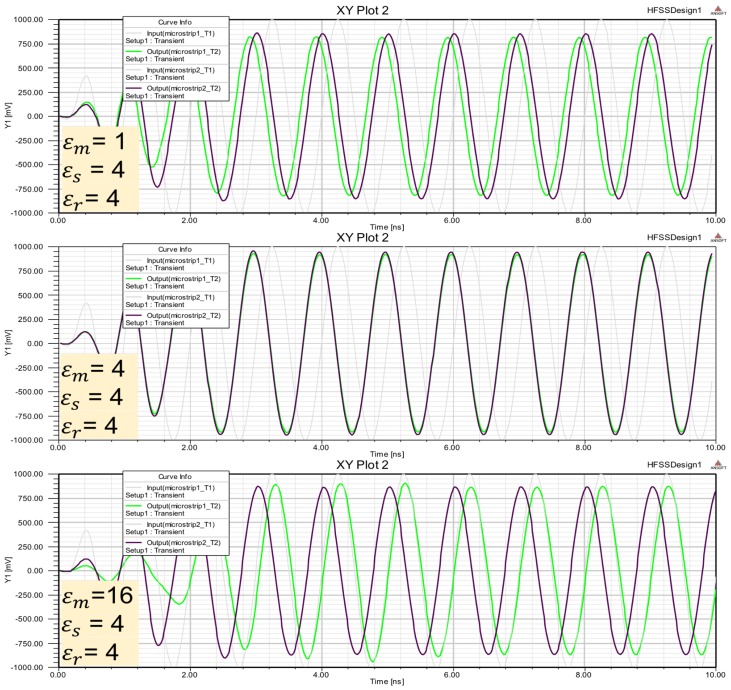
Microstrip line simulation with reference and substrate permittivity $\epsilon_{r}=4$. Different permittivity medium $\epsilon_{m}$ are used to obtain phase shift on microstrip terminals.

Simulations in HFSS software is shown in Figure 12.

### 6.2. Differential Measure Based in Electrical Field

A sinusoidal signal (Asinωt) is considered. This signal propagates along the transmission line in order to determine electrical fields on the microstrip lines. Signal propagates along *x*:

(17) $$E\left(x,t\right)=E_{0}e^{-\alpha x}\cos\left(\omega t-\beta x\right)$$

where

(18) $$\alpha =\omega \sqrt{\frac{\mu \epsilon }{2}\left[\sqrt{1+{\left(\frac{\sigma }{\omega \epsilon }\right)}^{2}}-1\right]}$$

(19) $$\beta =\omega \sqrt{\frac{\mu \epsilon }{2}\left[\sqrt{1+{\left(\frac{\sigma }{\omega \epsilon }\right)}^{2}}+1\right]}$$

In dilectric medium σ<<ωϵ, substituting these in Equations (18), (19) and simplifying:

(20) $$\begin{array}{c} \alpha =0\\ \beta =\omega \sqrt{\mu \epsilon_{eff}} \end{array}$$

Considering dielectric medium, differential measures are:

(21) $$d_{mr}=\Delta E=E_{0}\cos\left(\omega t-\omega \sqrt{\mu \epsilon_{meff}}x\right)-E_{0}\cos\left(\omega t-\omega \sqrt{\mu \epsilon_{reff}}x\right)$$

**Figure 13:**
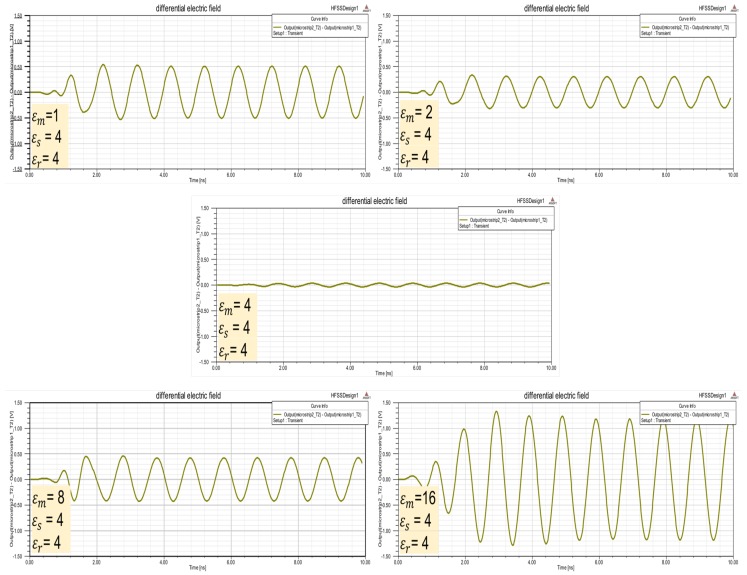
Microstrip line simulation with reference and substrate permittivity $\epsilon_{r}=4$. Different permittivity medium $\epsilon_{m}$ are used to obtain differential electric field on microstrip terminals.

Using HFSS software and construction shown in Figure 11, differential electric field obtained in $d_{mr}$ is in Figure 13. This table differential measurements confirm that when the unknown permittivity ($\epsilon_{m}$) is different to reference permittivity ($\epsilon_{r}$) electric field increases.

### 6.3. Microstrip Sensors

**Figure 14:**
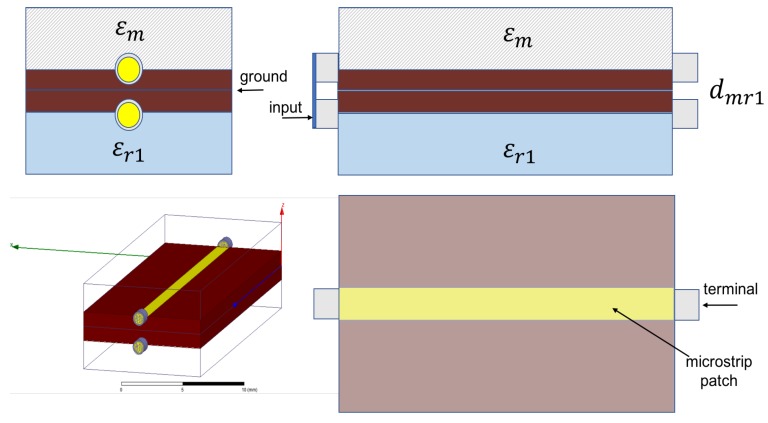
Microstrip line used as a sensor to detect permittivity levels of unknown material. Microstrip patch can have different dimensions and forms.

**Figure 15:**
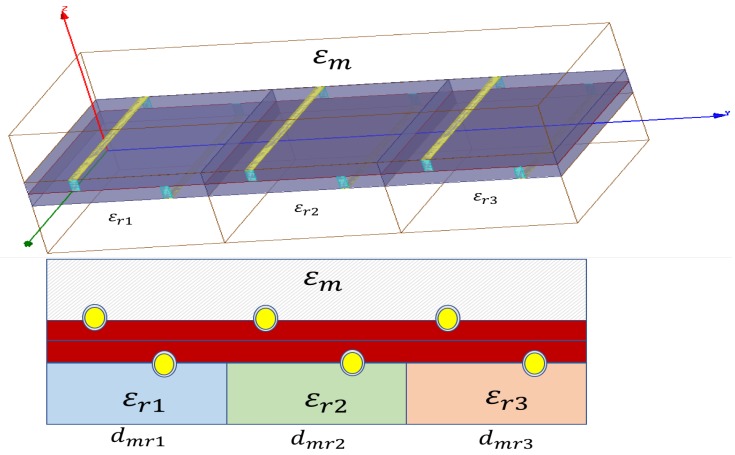
Configuration of a sensor microstrip that detect low, medium or high permittivity levels in applications where permittivity must be detected.

Differential electric field and phase shift simulations confirm potential use of sensors based on microstrip lines (Figure 14). Permittivity of an unknown material can be characterized using microstrip lines configuration. Accuracy values depend on different factors that must be considered (temperature, connection terminals, materials, etc.) and it is not easy to obtain. Nevertheless, reference permittivities can be used to detect if unknown material has the same permittivity or approximate. In these cases detector sensors have easy applicability. An example of this functionality is simulated using a microstrip formed by three modules (Figure 15). The goal is to detect if material permittivity is between minimum and maximum local values, and if it is near to the medium value. This example is useful when permittivity is variable over time (biological tissues) and it is necessary to detect limit values.

Electric fields and phase shift sign obtained in Table 5 provide data to design an heuristic model based in rules like:

$if\;\left(d_{mr}\;\left[condition\right]\;AND\;ph_{{sh}_{mr}}\;\left[condition\right]\right)\;then\;\epsilon_{m}\approx \left[value\right]$

Analyzing numeric results in multi reference microstrip simulation, some rules have been defined.

### 6.4. Simulation Platform and Other Electromagnetic Effects (Dielectric Losses and Materials)

This work analyze a new way to take advantage of current technologies (microstrip, stripline transmission, etc.) proposing a method to design sensors using configurations that measure signal differences. Hardware devices that produce and measure those differences must be adapted or built. This work validates this new method using a software platform that reproduce real conditions: ANSYS HFSS. This software is an industry standard for simulating high-frequency electromagnetic fields. Its gold-standard accuracy, advanced solvers and high-performance computing technologies make it an essential tool for researchers and engineers tasked with executing accurate and rapid test and design in high-frequency and high-speed electronic devices and platforms. HFSS offers state-of the-art solver technologies based on finite element, integral equation, asymptotic and advanced hybrid methods to solve a wide range of microwave, RF and high-speed digital applications [32]. Other effects like dielectric losses can be used to calculate material permittivity. If substrate used as a reference produce different losses than the unknown material substrate then the differences measured in receiver can be used in differential functions to determinate unknown material.

The simulation method is tested using relative dielectric constant of air and solids materials in range ϵ=[1,20], without conductivity (ρ=0). For other materials with more dielectric constant and conductivity, differences obtained will be more measurable. Reference material has ϵ=4,4.4,10,20 values. These values represent solids with reference permittivity known. In real development, references are known and can be different.

First, potential differences are theoretically calculated, using this model, to analyze measurable signals in the receiver. Finally, sensors will be built to take advantage of these differences.

## 7. Conclusions

Medium parameters like constant dielectric can be characterized using electromagnetic waves with different frequencies. Wave transmissions depends on the frequencies and the medium. Taking advantage of these interactions differential measures can be used to detect medium characteristic in microstrip sensors configuration. Different reference mediums and different frequencies can be used in the model proposed. Analysis and simulation results using differential measures in microstip configuration offer the following conclusions:

- In microstrip configuration (Figure 9 and Figure 14) differential measures ($d_{mr}$) depend on unknown material permittivity ($\epsilon_{m}$) and substrate design ($\epsilon_{r}$,$\epsilon_{r}$, $\epsilon_{d}$, *w* and *h*).
- Differential measures have positive sign or negative sign depending on whether the permittivity $\epsilon_{m}$ is greater or less (Table 4 and Figure 12).
- If unkown material permittivity $\epsilon_{m}$ is equal or similar to reference permittivity $\epsilon_{r}$, differential measures are minimum (Figure 13).
- Level detectors can be built using differential measures on microstrip configuration (Table 5).
- Multi reference materials can be used in substrate configuration to build levels detectors (Figure 15).
- Heuristic rules can be processed to detect levels exceeding limit values (Table 6).
- Materials permittivity could be characterized using microstrips configuration with reference substrate and adapted dimensions.

With these initial conclusions the use of differential method on microstip configuration proposed have different applications: (1) applications where maximum, normal or minimum permittivity levels must be detected and (2) applications where an unknown material permittivity must be characterized or detect. This microstrip configuration is proposed for future works in sensors design to analyze levels of dielectric properties of the skin and underlying tissue (blood glucose-levels have a maximun, medium and minimun levels that must to be detected). In this work differential measure is used with different reference mediums ($\epsilon_{r}$) to characterize unknown medium ($\epsilon_{m}$). The model will be expanded and completed, in future works, using multi-frequency waves, following the theoretic assumptions: differential measures, different characteristic mediums and multi-frequency waves.

## Figures and Tables

**Figure 7:**
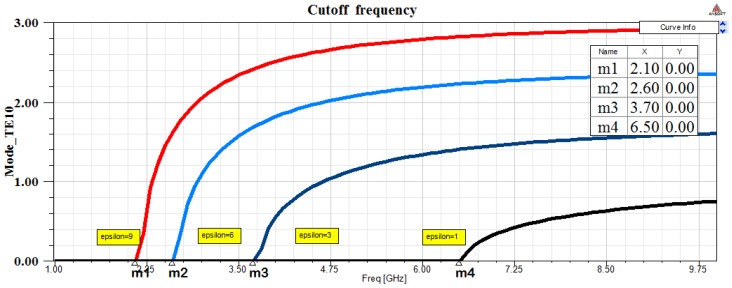
Frequency response in rectangular wave guide with different dielectric ($\epsilon_{r}$) materials. Simulation using HFSS software is performed. Differences in cutoff frequencies ($m_{i}-m_{j}=fc_{i}-fc_{j}=\Delta_{f}$) are obtained to calculate unknown $\epsilon_{r}$.

**Table 1:** Theoretical differential method proposed.

Medium is Represented by m Parameters in $M=M(p_{1},\ldots ,p_{m})$ Functions
**IF** Ψ is a measurable magnitude (speed propagation, time, amplitude, phase of arrival) which represents the interaction: wave (*E*) - medium (*M*), and the medium *M* has EM medium parameters ($p_{1},\ldots ,p_{m}$)
**THEN:** measurable magnitude Ψ in the receiver is a function Ψ=Ψ(E,M)
**IF** differences ($\Psi_{i}-\Psi_{j}$) are measured in the receiver **THEN:** different equations can be combined to calculate medium parameters ($p_{1},\ldots ,p_{m}$).
**1. IF** different frequencies are used in the interaction **THEN:** the equations system formed is:
$\Psi_{i}-\Psi_{j}=\Delta_{\Psi ij}=\Psi_{i}\left(E_{i},p_{1},\ldots ,p_{m}\right)-\Psi_{j}\left(E_{j},p_{1},\ldots ,p_{m}\right)$
($p_{1},\ldots ,p_{m}$) → are the unknown medium parameters
$E_{i}$ and $E_{j}$ → are EM waves at frequencies *i* and *j*
$\Delta_{\Psi ij}$ → are the differential measure obtained in receiver device
For n+1 frequencies there are *n* differential measures $\Delta_{\Psi ij}$ in receiver device.
**2. IF** only a frequency is used **THEN:** differential measures can be obtained with known medium parameters used as references. The same EM wave (*E*) is transmitted through a known reference medium ($E,p_{ref1},\ldots ,p_{refm}$) and through the unknown medium ($E,p_{1},\ldots ,p_{m}$). The equations are: $\Psi_{i}-\Psi_{j}=\Delta_{\Psi ij}=\Psi_{i}\left(E,p_{ref1},\ldots ,p_{refm}\right)-\Psi_{j}\left(E,p_{1},\ldots ,p_{m}\right)$

**Table 2:** Simulation measures (in time domain) obtained in three different materials. Simulated and theoretical results are compared. Time differences are obtained in waveguide transmission (shown in Figure 5).

$m_{2}-m_{1}=\Delta_{t}$	Simulated Result	Theoretical Result	Error %
0.0778ns	$\epsilon_{r}=3.16$	$\epsilon_{r}=3$	5.3
0.1493ns	$\epsilon_{r}=6.2$	$\epsilon_{r}=6$	3.3
0.2053ns	$\epsilon_{r}=9.32$	$\epsilon_{r}=9$	3.5

**Table 3:** Simulation measures (in frequency domain) obtained in three different materials. Cutoff frequency differences are obtained in waveguide transmission (shown in Figure 7). Theoretical and simulated measures are compared.

$m_{i}-m_{j}=fc_{i}-fc_{j}=\Delta_{f}$	Simulated Result	Theoretical Result	Error %
2.8 GHz	$\epsilon_{r}=3.2$	$\epsilon_{r}=3$	3.3
3.9 GHz	$\epsilon_{r}=6.2$	$\epsilon_{r}=6$	3.3
4.4 GHz	$\epsilon_{r}=9.3$	$\epsilon_{r}=9$	6.7

**Table 4:** Differential phase (rad) at the end of the line. Reference permittivity used is $\epsilon_{reff}=4$.

$\epsilon_{\mathrm{meff}}=1$	$\epsilon_{\mathrm{meff}}=2$	$\epsilon_{\mathrm{meff}}=3$	$\epsilon_{\mathrm{meff}}=4$	$\epsilon_{\mathrm{meff}}=5$	$\epsilon_{\mathrm{meff}}=6$	$\epsilon_{\mathrm{meff}}=7$	$\epsilon_{\mathrm{meff}}=8$
0.523599	0.306717	0.140298	0	−0.123605	−0.235352	−0.338115	−0.433763
1.0472	0.613434	0.280596	0	−0.24721	−0.470705	−0.676229	−0.867527
1.5708	0.920151	0.420894	0	−0.370815	−0.706057	−1.01434	−1.30129
2.0944	1.22687	0.561191	0	−0.49442	−0.94140	−1.35246	−1.73505
2.61799	1.53359	0.701489	0	−0.618025	−1.17676	−1.69057	−2.16882
3.14159	1.8403	0.841787	0	−0.741629	−1.41211	−2.02869	−2.60258
3.66519	2.14702	0.982085	0	−0.865234	−1.64747	−2.3668	−3.03634

**Table 5:** Numeric results of simulation realized using configuration shown in Figure 15. $d_{mr}$ represents differential electric field in *V* obtained in terminals. $sh_{mr}$ represents the phase shift sign obtained on electric field in terminals and $T_{sh_{mr}}$ is time shift in ns. Sinusoidal signal with f=1 GHz is used in this simulation.

	$\epsilon_{r1}=4.4$	$\epsilon_{r2}=10$	$\epsilon_{r3}=20$	$\epsilon_{r1}=4.4$	$\epsilon_{r2}=10$	$\epsilon_{r3}=20$	$\epsilon_{r1}=4.4$	$\epsilon_{r2}=10$	$\epsilon_{r3}=20$
$\epsilon_{m}$	$d_{\mathrm{mr}1}$	$d_{\mathrm{mr}2}$	$d_{\mathrm{mr}3}$	${\mathrm{sh}}_{\mathrm{mr}1}$	${\mathrm{sh}}_{\mathrm{mr}2}$	${\mathrm{sh}}_{\mathrm{mr}3}$	$T_{{\mathrm{sh}}_{\mathrm{mr}1}}$	$T_{{\mathrm{sh}}_{\mathrm{mr}2}}$	$T_{{\mathrm{sh}}_{\mathrm{mr}3}}$
1	0.6	0.8	>1	-	-	-	0.1	0.18	0.3
2	0.35	0.7	>1	-	-	-	0.05	0.1	0.2
4.4	0.1	0.4	1	+	-	-	0.01	0.05	0.15
8	0.3	0.15	0.75	+	-	-	0.04	0.08	0.1
10	0.5	0.1	0.6	+	+	-	0.1	0.02	0.1
15	1.0	0.5	0.3	+	+	-	0.2	0.1	0.08
20	>1	>1	0.1	+	+	+	0.3	0.1	0.01
30	>1	>1	>1	+	+	+	>1 ns	0.9	0.8

**Table 6:** Rules Configuration of a sensor microstrip that detect low, medium or high permittivity levels in applications where permittivity must be detected.

$\epsilon_{m}$	($if\;{sh}_{mr1}\;in\;{time}_{i}\neq \;{sh}_{mr1}\;in\;{time}_{i+1}\;)\;then$
crosses	$if\;{sh}_{mr1_{ti}}=-\;then\;\epsilon_{m}\in \left[\epsilon_{max},\epsilon_{max}\right]\;in\;{time}_{i+1}$
minimum level	$if\;{sh}_{mr1_{ti}}=+\;then\;\epsilon_{m}\notin \left[\epsilon_{max},\epsilon_{max}\right]\;in\;{time}_{i+1}$
$\epsilon_{m}$	$(if\;{sh}_{mr3}\;in\;{time}_{i}\neq \;{sh}_{mr3}\;in\;{time}_{i+1}\;)\;then$
crosses	$if\;{sh}_{mr3_{ti}}=+\;then\;\epsilon_{m}\in \left[\epsilon_{max},\epsilon_{max}\right]\;in\;{time}_{i+1}$
maximum level	$if\;{sh}_{mr3_{ti}}=-\;then\;\epsilon_{m}\notin \left[\epsilon_{max},\epsilon_{max}\right]\;in\;{time}_{i+1}$
	$if\;d_{mr1}\approx 0\;then\;\epsilon_{m}\to \epsilon_{r1}$
$\epsilon_{m}\in \left[\epsilon_{min},\epsilon_{max}\right]$	$if\;d_{mr2}\approx 0\;then\;\epsilon_{m}\to \epsilon_{r2}$
	$if\;d_{mr3}\approx 0\;then\;\epsilon_{m}\to \epsilon_{r3}$
